# The NF-κB subunit c-Rel regulates Bach2 tumour suppressor expression in B-cell lymphoma

**DOI:** 10.1038/onc.2015.399

**Published:** 2015-11-02

**Authors:** J E Hunter, J A Butterworth, B Zhao, H Sellier, K J Campbell, H D Thomas, C M Bacon, S J Cockell, B E Gewurz, N D Perkins

**Affiliations:** 1 Institute for Cell and Molecular Biosciences (ICaMB), Newcastle University Medical School, Newcastle Upon Tyne, UK; 2 Brigham and Women's Hospital, Boston, MA, USA; 3 The Beatson Institute for Cancer Research, Glasgow, UK; 4 Northern Institute for Cancer Research, Newcastle Upon Tyne, UK; 5 Bioinformatics Support Unit, Faculty of Medical Sciences, Newcastle University, Newcastle Upon Tyne, UK

## Abstract

The REL gene, encoding the NF-κB subunit c-Rel, is frequently amplified in B-cell lymphoma and functions as a tumour-promoting transcription factor. Here we report the surprising result that *c-rel–/–* mice display significantly earlier lymphomagenesis in the c-Myc driven, E*μ-Myc* model of B-cell lymphoma. c-Rel loss also led to earlier onset of disease in a separate TCL1-Tg-driven lymphoma model. Tumour reimplantation experiments indicated that this is an effect intrinsic to the E*μ-Myc* lymphoma cells but, counterintuitively, *c-rel–/–* E*μ-Myc* lymphoma cells were more sensitive to apoptotic stimuli. To learn more about why loss of c-Rel led to earlier onset of disease, microarray gene expression analysis was performed on B cells from 4-week-old, wild-type and *c-rel–/–* E*μ-Myc* mice. Extensive changes in gene expression were not seen at this age, but among those transcripts significantly downregulated by the loss of c-Rel was the B-cell tumour suppressor BTB and CNC homology 2 (Bach2). Quantitative PCR and western blot analysis confirmed loss of Bach2 in c-Rel mutant E*μ-Myc* tumours at both 4 weeks and the terminal stages of disease. Moreover, Bach2 expression was also downregulated in *c-rel–/–* TCL1-Tg mice and RelA Thr505Ala mutant E*μ-Myc* mice. Analysis of wild-type E*μ-Myc* mice demonstrated that the population expressing low levels of Bach2 exhibited the earlier onset of lymphoma seen in *c-rel–/–* mice. Confirming the relevance of these findings to human disease, analysis of chromatin immunoprecipitation sequencing data revealed that Bach2 is a c-Rel and NF-κB target gene in transformed human B cells, whereas treatment of Burkitt's lymphoma cells with inhibitors of the NF-κB/IκB kinase pathway or deletion of c-Rel or RelA resulted in loss of Bach2 expression. These data reveal a surprising tumour suppressor role for c-Rel in lymphoma development explained by regulation of Bach2 expression, underlining the context-dependent complexity of NF-κB signalling in cancer.

## Introduction

The tumour-promoting role of the NF-κB pathway is well established and results from its ability to regulate the expression of genes involved in multiple aspects of cancer cell biology.^1^ This is also true in haematological malignancies^2^ and in several B-cell lymphoma types, such as activated B-cell-like-diffuse large B-cell lymphomas,^3^ primary mediastinal large B-cell lymphoma^4, 5^ and classical Hodgkin lymphoma^6^ NF-κB activity is required for survival and proliferation. However, the contribution of individual NF-κB subunits is generally not known. In particular, whereas NF-κB subunits have been reported to exhibit characteristics of tumour suppressors *in vitro*,^1^ it has not been investigated whether these properties have relevance to lymphoma development *in vivo*.

There are five NF-κB subunits in mammalian cells, RelA/p65, RelB, c-Rel, p50/p105 (NF-κB1) and p52/p100 (NF-κB2). RelA and c-Rel function as effector subunits for the IκB kinase β-dependent, canonical NF-κB pathway.^7^ Of these NF-κB subunits, c-Rel is most closely associated with lymphoma and was first identified as the cellular homologue of the avian Rev-T retroviral oncogene v-Rel.^8, 9, 10^ c-Rel is ubiquitously expressed in B cells regardless of developmental stage, although the highest levels are observed in mature B cells.^11, 12, 13^ *c-rel* knockout mice developed normally with no effects on B-cell maturation but do exhibit some immunological defects, including reduced B-cell proliferation and activation, abnormal germinal centres and reduced number of marginal zone B cells.^14, 15, 16, 17^

c-Rel is distinct from other NF-κB family members in its ability to transform chicken lymphoid cells *in vitro*.^8, 18, 19, 20^ Moreover, genomic and cytogenetic studies of human lymphomas have shown gains of chromosome 2p13, which encodes the *REL* gene. Amplifications and gains of *REL* have been detected in ~50% of HL^21, 22, 23^ and 10–25% or 50% in two studies of primary mediastinal large B-cell lymphoma.^4, 24^ *REL* has also been identified as a susceptibility locus for HL,^25^ whereas c-Rel nuclear localisation has been identified as a poor prognostic factor in both activated B-cell-like- and germinal centre B-cell-like-diffuse large B-cell lymphomas.^26^

Despite this, relatively little is known about the role of c-Rel or other NF-κB subunits in c-Myc-driven lymphomas. However, a recent study of Myc-driven B-cell lymphoma in mice revealed a tumour suppressor role for RelA.^27^ Here, short hairpin RNA silencing of RelA did not affect progression of established lymphomas, but after cyclophosphamide treatment its loss resulted in chemoresistance as a consequence of impaired induction of senescence.^27^ Similarly, NF-κB was required for both therapy-induced senescence and resistance to cell death in the E*μ-Myc* mouse model of B-cell lymphoma upon expression of a degradation-resistant form of IκBα.^28^ c-Myc can also inhibit expression of NF-κB2, and loss of this NF-κB subunit in the E*μ-Myc* mouse model resulted in moderately earlier onset of disease as a consequence of impaired apoptosis.^29^ By contrast, deletion of NF-κB1 displayed no effects on E*μ-Myc* lymphoma development.^30^ These results imply a more complicated role for NF-κB in Myc-driven lymphoma, with both tumour-promoting and -suppressing functions being reported, although any role for c-Rel has not been established.

Here, we have investigated the role of c-Rel in mouse models of B-cell lymphomagenesis. We demonstrate that, opposite to the expected result, *c-rel–/–* E*μ-Myc* and TCL1-Tg mice exhibit earlier onset of lymphoma and that this result can be explained by c-Rel-dependent regulation of the B-cell tumour suppressor BTB and CNC homology 2 (Bach2).

## Results

### NF-κB is active in E*μ-Myc*-derived lymphoma

**Figure 1:**
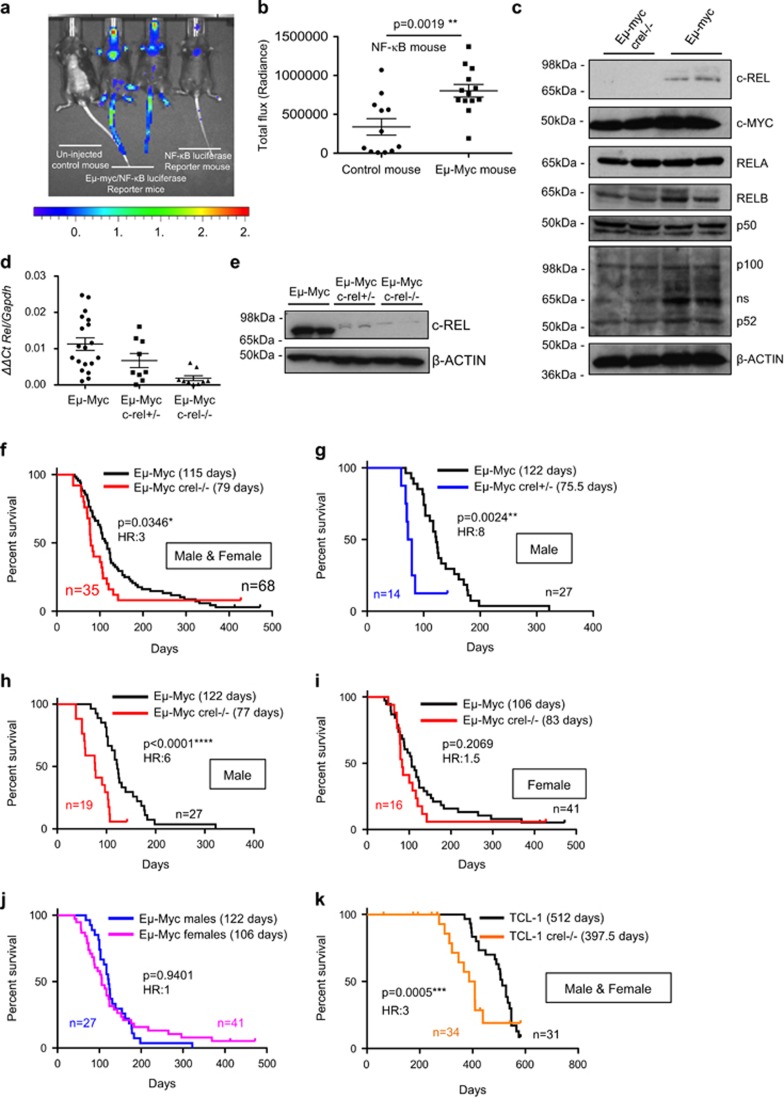
c-Rel functions as a tumour suppressor in E*μ-Myc*-driven B-cell lymphoma in mice. (**a**) Representative image of *in vivo* NF-κB bioluminescence (radiance) of age-matched littermates of *NF-*κ*B-Luc* and E*μ-Myc/NF-*κ*B-Luc* mice. Eight-week-old mice underwent *in vivo* imaging using the IVIS Spectrum system (Perkin Elmer, Beaconsfield, UK) after being intraperitoneally administration with 150 mg/kg VivoGlo d-luciferin (Promega, Southampton, UK) dissolved in sterile phosphate-buffered saline. Ten-min post-d-luciferin-administration, mice were imaged using a photon emission over 5 min, under isoflurane anaesthesia. Luminescence was seen in the thymic area and also in the tails and other exposed regions of the E*μ-Myc/NF-*κ*B-Luc* mice, the latter likely due to a higher number of circulating lymphocytes with increased NF-κB activity. (**b**) Quantification of NF-κB bioluminescence (radiance) of thymic regions in *NF-*κ*B-Luc* (*n*=12) and E*μ-Myc/NF-*κ*B-Luc* (*n*=13) mice. Bioluminescence was quantified using the Living Image software version 4.3.1 (Perkins Elmer) and region of interest tool. Data shown as mean±s.e.m., **P<0.01, unpaired Student's *t*-tests. For all tests, where appropriate, analyses were undertaken to test for normal distribution. (**c**) Western blot analysis of the NF-κB subunits, c-REL, RELA, RELB, p100/p52 and p50 together with c-MYC in extracts prepared from E*μ*-Myc and E*μ-Myc/c-rel–/–* mouse tumorigenic spleens. Whole-cell extracts were prepared from E*μ-Myc* or E*μ-Myc/c-rel–/–* tumour cell suspensions. Cell pellets were washed with ice-cold phosphate-buffered saline and lysed using PhosphoSafe Extraction Reagent (Merck Millipore, Watford, UK). Antibodies used were c-Rel (sc-71 Santa Cruz, Insight Biotechnology, Wembley, UK), c-Myc (sc-42 Santa Cruz), RelA (sc-372 Santa Cruz), RelB (4954 Cell Signaling, Hitchin, UK), p50 (06-886 Merck Millipore), p100/p52 (sc-848 Santa Cruz) and β-Actin (A5441 Sigma-Aldrich, Gillingham, UK). (**d**) Quantitative-PCR analysis showing relative *Rel* expression in end-stage tumorigenic spleens from E*μ-Myc* (*n*=20), E*μ-Myc*/*c-rel+/–* (*n*=12) and E*μ-Myc*/*c-rel–/–* (*n*=11) mice. Data shown as mean±s.e.m., each point is an individual mouse. (**e**) Western blot analysis of c-REL levels in tumorigenic spleens from E*μ-Myc,* E*μ-Myc*/*c-rel+/–* and E*μ-Myc/c-rel–/–* mice. (**f–j**) Reduced survival of E*μ-Myc*/*c-rel+/–* and E*μ-Myc/c-rel–/–* mice. Kaplan–Meier plots showing survival curves for E*μ-Myc* and (**f**) E*μ-Myc/c-rel–/–* mice, (**g**) E*μ-Myc/c-rel+/–* male mice, (**h**) E*μ-Myc/c-rel–/–* male mice, (**i**) E*μ-Myc/c-rel–/–* female mice and relative survival of male versus female E*μ-Myc* mice is shown in (**j**). *P*-values (Mantel–Cox test) and hazard ratios are shown. (**k**) Kaplan–Meier plot showing reduced survival of TCL1*/c-rel–/–* mice. Animal handling, husbandry and experimentation were undertaken in compliance with UK Home Office regulations under project licences and approved by the local ethical review committee. All mice used in these experiments were on C57BL/6 background and bred at the Comparative Biology Centre, Newcastle University. *c-rel–/–* mice were provided by Dr Fiona Oakley (Newcastle University). NF-κB-luc (NF-κB-Luc*–*/+) reporter mice were a gift from Professor Matthew Wright (Newcastle) and originated in the laboratory of Professor Harald Carlsen (Norwegian University of Life Sciences). E*μ-Myc* and TCL1-Tg mice were purchased from The Jackson Laboratory (Bar Harbor, ME, USA). E*μ-Myc/c-rel+/–* offspring were generated by mating *c-rel–/–* female mice with E*μ-Myc* male mice. E*μ-Myc/c-rel–/–* mice were then generated by crossing E*μ-Myc/c-rel+/–* males with *c-rel–/–* female mice. In TCL1-Tg mice, a human *TCL1* coding sequence is expressed from a B29 minimal promoter, coupled with the IgH intronic enhancer resulting in B- and T-cell expression. TCL1*/c-rel–/–* offspring were generated as for E*μ-Myc* by mating *c-rel–/–* female mice with TCL1-Tg male mice. All mice were designated to an experimental group-dependent on their strain and no blinding was undertaken during analysis. For survival analysis, mice were monitored daily and were killed at predetermined end points, defined as the animal becoming moribund, losing bodyweight/condition and/or having palpable tumour burden at any lymphoid organ site, at which point animals underwent necropsy. Kaplan–Meier survival curves were drawn using GraphPad Prism (Version 5.0, GraphPad Software, La Joll, CA, USA).

To determine if there are significant levels of NF-κB activity in Myc-driven B-cell lymphoma, with the potential to affect disease driven by this oncogene, we crossed *3 ×* κ*B-luc (NF-*κ*B-Luc)* reporter mice onto E*μ-Myc* transgenic mice, allowing *in vivo* visualisation of NF-κB activity.^31^ The median onset of aggressive lymphoma in E*μ-Myc* mice is between the ages of 3 and 6 months but they exhibit the hallmarks of Myc overexpression by 4 weeks.^32^ This analysis revealed significantly higher levels of NF-κB activity in E*μ-Myc* mice at 8 weeks of age, in lymphoid organ sites, including mesenteric/inguinal lymph nodes and thymus (Figures 1a and b).

### Loss of c-Rel results in earlier onset of E*μ-Myc*-driven lymphoma

To investigate the role of c-Rel in MYC-induced lymphomagenesis, E*μ-Myc/c-rel–/–* mice were generated. Western blot analysis confirmed no significant effects on the other NF-κB subunits or c-Myc in splenic tumour B cells, although slightly lower levels of the non-canonical NF-κB subunits p52 and RelB were found in *c-rel–/–* cells (Figure 1c). E*μ-Myc/c-rel+/–* mice, despite having intermediate levels of c-Rel mRNA (Figure 1d), had almost no detectable c-Rel protein in E*μ-Myc* lymphoma cells (Figure 1e).

Given the known tumour-promoting role of c-Rel in B-cell lymphoma, we were surprised to find that E*μ-Myc/c-rel–/–* mice had a significantly shorter overall survival (median survival 79 days) than E*μ-Myc* mice (median survival 115 days; Figure 1f). Earlier onset of disease was also seen in heterozygote E*μ-Myc/c-rel+/–* male mice (median onset 75.5 days; Figure 1g). Although survival times of male and female E*μ-Myc/c-rel–/–* mice were similar (77 vs 83 days, respectively; Figures 1h and i), this effect appeared more pronounced in male c-Rel mutant mice due to gender differences in wild-type E*μ-Myc* mice (122 days in males vs 106 days in females), although this difference was not statistically significant (Figure 1j).

To determine if earlier onset of disease could be seen in other lymphoma models, we generated *c-rel–/–* strains of pE*μ*-B29-TCL1 (TCL1-Tg) transgenic mice.^33^ These mice exhibit slower disease progression than in the E*μ-Myc* model and in our experiments many mice developed tumours at non-lymphoid sites (not shown). Nonetheless, *c-rel–/–* mice again displayed significantly reduced survival relative to wild-type TCL1 mice, confirming that this effect is not restricted to the E*μ-Myc* model (Figure 1k).

### Reimplanted E*μ-Myc* tumours grow equally well in wild-type and *c-rel–/–* mice

**Figure 2:**
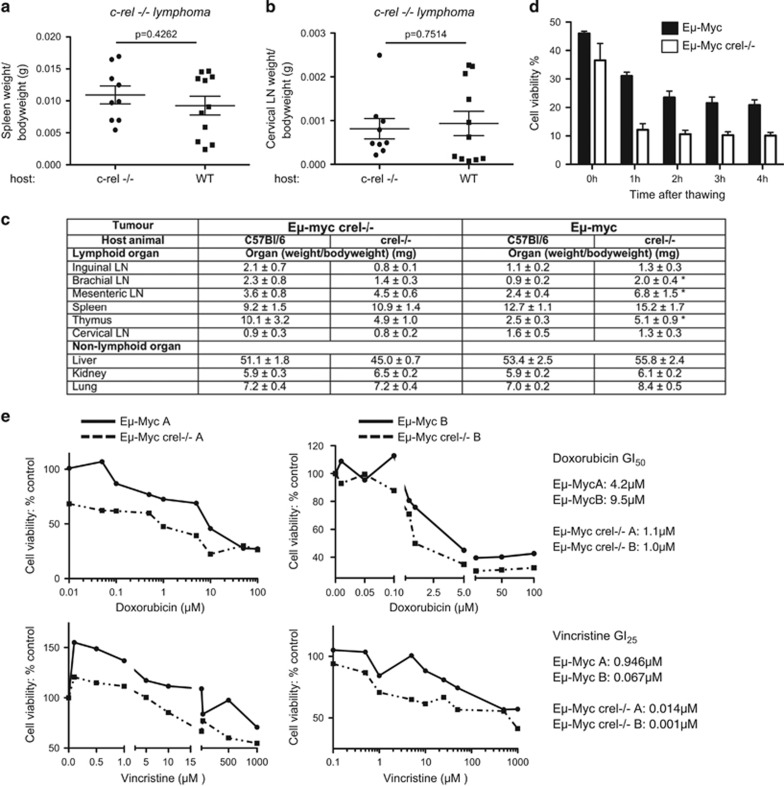
E*μ-Myc/c-rel–/–* tumours grow equally well in wild-type and *c-rel–/–* mice and are more sensitive to apoptotic stimuli. (**a**, **b**) Reimplanted E*μ-Myc/c-rel–/–* tumours grow equally well in wild-type and *c-rel–/–* mice. Lymph node tumours derived from three different E*μ-Myc/c-rel–/–* mice were reimplanted in parallel into either three wild-type (C57Bl/6) or three *c-rel–/–* host mice. Four weeks after implantation, the mice were killed and tumour sizes at different sites were assessed. Data shown here are from the spleen (**a**) and cervical lymph nodes (**b**). Data representing mean±s.e.m. and *P*-values were calculated using Student's unpaired *t*-tests. (**c**) Tumour burden in lymphoid organs (weight of organ/bodyweight of animal in gram) following reimplantation of either Eμ-Myc or Eμ-Myc c-rel*–/–* tumour cells into either C57Bl/6 or c-rel*–/–* mice. Data shown are the means of three independent tumours each implanted into three mice±s.e.m. *P<0.05 in an unpaired Student's *t*-test, but otherwise there were no significant differences between tumour burden in wild-type and c-rel knock-out animals at any of the sites assessed. (**d**) Cell viability of Eμ-Myc and Eμ-Myc/c-rel*–/–* tumour cells grown *ex vivo*. Cell viability was measured using the trypan blue exclusion assay over a 4-h period after freeze thawing. (**e**) E*μ-Myc/c-rel–/–* tumour cells are more sensitive to apoptotic stimuli. Freshly isolated E*μ-Myc* or E*μ-Myc/c-rel–/–* lymph node tumour cells (5 × 10^5^ per well) were seeded into 96-well plates. Increasing concentrations of the chemotherapeutic agents, doxorubicin (Sigma-Aldrich) or vincristine (Sigma-Aldrich) or solvent controls were added to three replicate wells. After 96 h, viability was quantified using the CellTiter96 AQ_ueous_ One Solution Cell Proliferation Assay (MTS; Promega), according to the manufacturer's instructions. Single-cell suspensions were prepared from tumour-bearing organs of E*μ-Myc* and E*μ-Myc/c-rel–/–* mice upon necropsy. These were then used for downstream analyses or frozen in 90% fetal bovine serum/10% dimethyl sulfoxide for long-term storage and transplantation. For reciprocal microenvironment experiments, 2 × 10^6^ E*μ-Myc/c-rel–/–* lymph node tumour cells from male mice were transplanted intravenously via the lateral tail vein into 8-week-old male C57BL/6 or *c-rel–/–* recipients. Mice were necropsied when they became moribund and the tumour burden assessed. C57BL/6 mice used for reimplantation studies were purchased from Charles River (Margate, UK).

These results revealed an apparent tumour suppressor role for c-Rel, but it was unclear if this resulted from an effect intrinsic to the tumour cells or from other effects of the *c-rel–/–* mice. Therefore, to investigate whether non-tumour cells in the wild-type and *c-rel–/–* mice might contribute to earlier onset of disease in c-Rel null mice, we performed a series of reciprocal tumour reimplantation studies. Tumours derived from either wild-type or *c-rel–/–* male E*μ-Myc* mice were transplanted into either C57Bl/6 or *c-rel–/–* male host mice. Importantly, whether the host mice were wild type or *c-rel–/–* did not affect the rate of *c-rel–/–* lymphoma growth (Figures 2a and c). A more mixed effect was seen with reimplanted wild-type E*μ-Myc* cells, where increased lymphoma growth was seen at some sites but not others in the *c-rel–/–* host mice (Figure 2c). Reimplanted *c-rel–/–* lymphomas were also slower to develop than wild type (~4 weeks vs 2 weeks) but this may reflect the reduced viability of E*μ-Myc/c-rel–/–* tumour cells after thawing frozen samples (Figure 2d). This analysis does not rule out a contribution from the non-tumour background in the development of E*μ-Myc* lymphoma in these mice. However, given that we saw no effects of the host animal on the growth of reimplanted *c-rel–/–* cells, we investigated if there were intrinsic differences between wild-type and *c-rel–/–* lymphoma cells.

### c-rel*–/–* B-cell lymphomas are more sensitive to apoptotic stimuli

c-Rel and the other NF-κB subunits can contribute towards tumorigenesis by inducing the expression of antiapoptotic genes^34^ and, consistent with this and the results in Figure 2d, we found that when cultured *ex vivo*, tumour cell isolates from E*μ-Myc/c-rel–/–* mice showed increased sensitivity to the R-CHOP therapy components doxorubicin and vincristine (Figure 2e). Therefore, E*μ-Myc/c-rel−/−* cells appear more prone to apoptosis when compared with their wild-type equivalents. These effects are consistent with the known antiapoptotic effects of c-Rel but did not explain the earlier onset of disease in c-Rel null mice.

### The tumour suppressor Bach2 is a c-Rel target gene

The p53 and ARF pathways are frequently disrupted in E*μ-Myc* lymphoma.^35^ However, we found that mRNA levels of p53 target genes, such as *Mdm2* and *Bax*, as well as the *CDKN2A* gene that encodes the ARF protein were similar across end-stage E*μ-Myc* and E*μ-Myc/c-rel–/–* tumour cells (not shown), suggesting that c-Rel loss does not lead to further disruption of these pathways. Moreover, no significant differences in *BCL2L1* mRNA, an NF-κB target gene that encodes the antiapoptotic protein Bcl-xL,^34^ were observed (not shown).

We therefore wanted to learn more about other changes in gene expression associated with the earlier onset of lymphoma in the E*μ-Myc/c-rel–/–* mice. Consequently, we decided to perform microarray-based genome-wide mRNA expression analyses on B cells from 4-week-old E*μ-Myc,* E*μ-Myc/c-rel+/–* and E*μ-Myc/c-rel–/–* mice.

**Figure 3:**
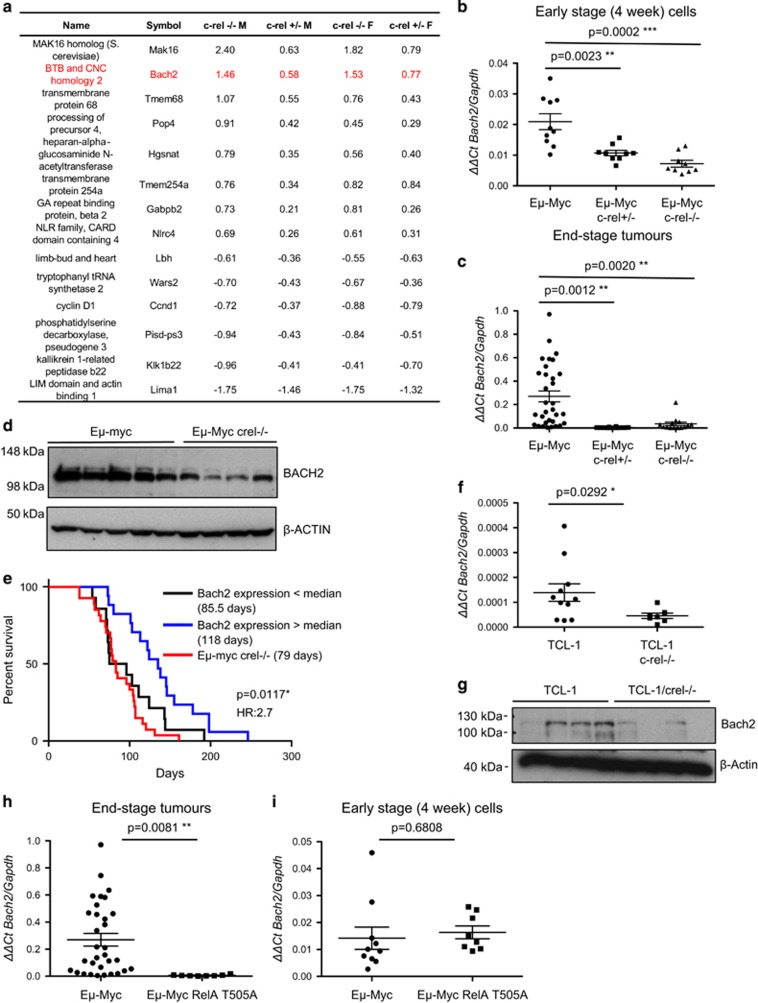
Expression of the B-cell tumour suppressor Bach2 is dependent on c-Rel in E*μ-Myc* lymphoma. (**a**) Table showing genes whose expression is regulated by c-Rel from microarray analysis of bone marrow-derived B cells from 4-week-old E*μ-Myc*, E*μ-Myc*/*c-rel+/–* and E*μ-Myc*/*c-rel–/–* mice. Fold changes shown are compared with equivalent wild-type cells and are in log2 (a positive number indicates higher expression in wild-type cells). Bone marrow-derived B cells were purified from 4-week-old *Eμ-Myc* or *Eμ-Myc/c-rel+/–, Eμ-Myc/c-rel+/–* mice using CD19 microbeads (MACS Miltenyi Biotec, Surrey, UK). Total B-cell RNA, purified using the PeqGold total RNA extraction kit (Peqlab, VWR, Lutterworth, UK), was then used for microarray analysis at Cambridge Genomic Services (University of Cambridge, Cambridge, UK) using the Illumina mouse WG-6 Expression BeadChip system (San Diego, CA, USA). These data were background corrected in Illumina GenomeStudio and subsequent analysis proceeded using the lumi and limma packages in R (Bioconductor, Seattle, WA, USA).^46, 47, 48^ Variant stabilisation transform and robust spline normalisation were applied in lumi. Differential expression was detected using linear models and empirical Bayes statistics in limma. A list of genes for each comparison was generated using a Benjamini–Hochberg false discovery rate-corrected *P*-value of 0.05 as a cutoff. (**b**, **c**) Confirmation that Bach2 mRNA levels are c-Rel regulated. Quantitative-PCR (q-PCR) showing relative *Bach2* expression in (**b**) bone marrow-derived B cells from E*μ-Myc* (*n*=10), E*μ-Myc*/*c-rel+/–* (*n*=9) and E*μ-Myc*/*c-rel–/–* (*n*=9) mice and (**c**) end-stage tumorigenic spleens from E*μ-Myc* (*n*=30), E*μ-Myc*/*c-rel+/–* (*n*=12) and E*μ-Myc*/*c-rel–/–* (*n*=11) mice. q-PCR was performed in triplicate on 20 ng cDNA (Reverse Transcriptase kit, Qiagen, Crawley, UK), using predesigned *Bach2* Quanititect Primer assays (Qiagen). Samples were run and analysed on a Rotor-gene Q system (Qiagen), using murine *Gapdh* primers as an internal control. All cycle threshold values were normalised to *Gapdh* levels using the Pfaffl method.^49^ Data represent mean±s.e.m. **P<0.01, ***P<0.001 (unpaired Student's *t*-test). (**d**) Bach2 protein levels are reduced in E*μ-Myc*/*c-rel–/–* mice. Whole-cell extracts were prepared from E*μ-Myc* or E*μ-Myc/c-rel–/–* tumourigenic spleens. Cell pellets were washed with ice-cold phosphate-buffered saline, and lysed using PhosphoSafe Extraction Reagent (Merck Millipore), according to the manufacturer's protocols. Western blot analysis was performed using antibodies to BACH2 (ab83364 Abcam, Cambridge, UK) or the loading control β-ACTIN (A5441 Sigma-Aldrich). (**e**) Low levels of Bach2 mRNA correlate with poor survival in wild-type E*μ-Myc* mice. Kaplan–Meier analysis of the survival of mice with below and above the median levels of Bach2 mRNA (from data in **c**). Also shown for comparison is the survival data from E*μ-Myc/c-rel–/–* mice shown in Figure 1f. (**f**) Bach2 mRNA levels are c-Rel regulated in TCL1-Tg mice. q-PCR showing relative *Bach2* expression in end-stage tumorigenic spleens from TCL1-Tg (*n*=11) and TCL1-Tg/*c-rel–/–* (*n*=7) mice. Data represent mean±s.e.m. *P<0.05. (**g**) Bach2 protein levels are reduced in TCL1/*c-rel–/–* mice. Whole-cell extracts were prepared from TCL1-Tg or TCL1*/c-rel–/–* tumourigenic spleens and western blot analysis was performed as indcated. (**h**, **i**) Low Bach2 mRNA levels in RelA T505A mice. q-PCR showing relative *Bach2* expression in (**h**) end-stage tumorigenic spleens from E*μ-Myc* (*n*=30) and E*μ-Myc*/*rela*^*T505A*^ (*n*=8) mice and (**i**) bone marrow-derived B cells from E*μ-Myc* (*n*=10) and E*μ-Myc*/*rela*^*T505A*^ (*n*=8) mice. Note, data from wild-type E*μ-Myc* mice are the same as shown in **c**. Data represent mean±s.e.m. **P<0.01 (unpaired Student's *t*-test). RelA T505A knock-in mice were generated by Taconic Artemis (Cologne, Germany) using C57Bl/6 ES cells.

Analysis of these microarray data identified a number of genes misregulated in E*μ-Myc/c-rel–/–* mice (Figure 3a). Of these, the loss of expression of Bach2 in c-Rel mutant mice was of particular interest. Bach2 is a lymphoid-specific transcription factor with a role in B-cell development^36^ and the response to oxidative stress.^37, 38^ Bach2 has also been identified as a tumour suppressor in acute lymphoblastic leukaemia.^39^ Importantly, quantitative PCR analysis confirmed that *Bach2* mRNA expression is lost in B cells from 4-week-old E*μ-Myc/c-rel+/–* and E*μ-Myc/c-rel–/–* mice (Figure 3b), and also from the tumours taken from mice killed with end-stage disease (Figure 3c). Bach2 protein levels were also significantly reduced in the E*μ-Myc/c-rel−/−* tumours (Figure 3d). Quantitative PCR also validated a number of other potential targets identified in the microarray, including Cyclin D1 and Lima1 (not shown). Although Bach2 levels were reduced in normal, untransformed B cells from *c-rel–/–* 4-week-old mice, this was not a statistically significant effect (not shown).

Although Bach2 mRNA levels are uniformly low in all E*μ-Myc/c-rel–/–* and *c-rel+/–* lymphoma samples analysed, we observed a wide range of *Bach2* mRNA expression in end-stage wild-type E*μ-Myc* tumours (Figure 3c). We were therefore interested in whether this would correlate with survival of these wild-type E*μ-Myc* mice. Significantly, we found that E*μ-Myc* mice with below-the-median level of *Bach2* mRNA displayed decreased survival, with a median survival of 85.5 versus 135 days for mice with high Bach2 levels (Figure 3e). Therefore, wild-type mice with reduced levels of Bach2 have a very similar pattern of lymphoma onset to that seen in the c-rel*–/–* mice, providing a potential mechanism that allows this NF-κB subunit to function as a tumour suppressor in this model of c-Myc-driven B-cell lymphoma (Figure 3e).

To determine the generality of these effects we also analysed Bach2 levels in the spleens of TCL1-Tg mice, where we observed a reduction in mRNA and protein levels (Figures 3f and g). Furthermore, in a separate NF-κB knock in mouse model, where the RelA subunit was engineered to contain a Thr505Ala mutation in its transactivation domain, a site previously shown to affect NF-κB function,^40^ loss of Bach2 expression was also seen in end-stage lymphoma cells (Figure 3h) but not in 4-week B cells from E*μ-Myc* mice (Figure 3i). The RelA T505A mouse will be described in more detail elsewhere.

**Figure 4:**
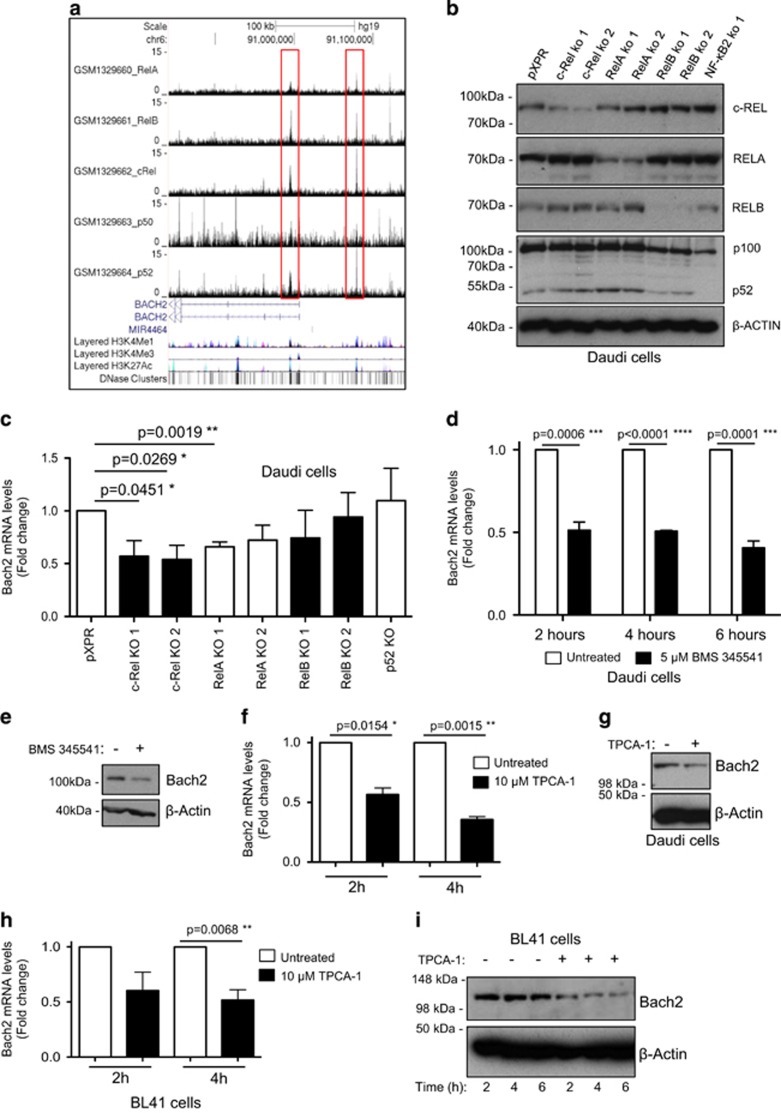
Bach2 is an NF-κB target gene on human B-cell malignancies. (**a**) ChIP-Seq data showing NF-κB subunit binding in the region of the human BACH2 gene in the Epstein–Barr-virus-transformed lymphoblastoid B-cell line (LCL) GM12878. ChIP-Seq data were extracted from a previously published analysis of the Epstein–Barr-virus-transformed LCL GM12878 using validated anti-RelA, RelB, c-Rel, p52 and p50 antibodies.^41^ Reads from all ChIP-Seq experiments were mapped to the hg19/GRCh37 build of the human genome using the UCSC genome browser. (**b**, **c**) c-Rel and RelA regulate *Bach2* mRNA levels in Daudi cells. In **b** western blot analysis shows depletion of NF-κB subunits in the Daudi Burkitt's lymphoma cell line using CRISPR/Cas9 mutagenesis. In **c** q-PCR shows relative *Bach2* expression in the Daudi cells with mutated NF-κB subunits. Data are obtained from separately derived pools of Daudi Cas9+ cells that express either a control single-guide RNA (sgRNA) against GFP (pXPR) or an sgRNA against the indicated NF-κB subunit. RNA or protein was extracted for either q-PCR (**b**) or western blot (**c**) analysis, as indicated. Daudi Cas9/CRISPR analysis: Daudi cells with stable Cas9 expression were derived as previously described.^50^ Briefly, Daudi cells with stable *Streptococcus pyogenes* Cas9 expression were established by infection with lentiviral transduction and blasticidin selection, using pLentiCas9-Blast (Addgene plasmid #52962). Cas9 activity was validated by transduction of the Daudi Cas9+ cells with a test lentivirus, which encodes a GFP and a sgRNA that targets GFP.^51^ The PXPR-011 plasmid (provided by John Doench, Broad Institute, Cambridge, MA, USA) was used to construct this test virus. Cas9 activity was evident in >85% of the selected Daudi cells by flow cytometry analysis (the residual 15% of cells that continue to express GFP may be cells where the non-homologous end-joining pathway correctly repaired the Cas9-induced DNA double-strand break).^51^ To obtain NF-κB subunit knockdown by CRISPR/Cas9 genome editing, the following sgRNAs were designed using CRISPRdirect (http://crispr.dbcls.jp/):^52^ RelA 5′-AGTCCTTTCCTACAAGCTCG-3′ and 5′-AGCTGATGTGCACCGACAAG-3′ RelB 5′-GGTCTGGCGACGCGGCGACT-3′ and 5′-AGCGGCCCTCGCACTCGTAG-3′ c-Rel 5′-AAATGTGAAGGGCGATCAGC-3′ and 5′-ATTGGGTTCGAGACAACAGG-3′ p52 5′-TAGGCTGTTCCACGATCACC-3′. Oligonucleotides were synthesized by Life Technologies (Paisley, UK), were individually cloned into the lentiGuide-Puro vector (Addgene plasmid #52963), according to the protocol from the Zhang laboratory (http://genome-engineering.org/),^53^ and were sequence verified. VSV-G pseudotyped lentiviruses encoding a sgRNA were produced in 293 T cells and used to transduce Daudi Cas9+ cells. Cells transduced with sgRNA-encoding lentivirus were selected by puromycin. (**d**–**g**) Treatment of the Daudi Burkitt's lymphoma cell line with the IκB kinase inhibitors BMS 345541 and TPCA-1 reduces BACH2 mRNA and protein levels. Daudi cells were treated with either 5 *μ*M BMS 345541 (Calbiochem, San Diego, CA, USA) or 10 *μ*M TPCA-1 (Calbiochem) for the times shown. RNA or protein was extracted for either q-PCR (**d**, **f**) or western blot (**e**, **g**) analysis using the Bach2 antibody, ABN171 (Merck Millipore). (**h**, **i**) Treatment of the BL41 Burkitt's lymphoma cell lines with the IκB kinase inhibitor TPCA-1 reduces BACH2 mRNA and protein levels. BL41 cells were treated with 10*μ*M TPCA-1 for the times shown. RNA or protein was extracted for either q-PCR (**h**) or western blot (**i**) analysis. q-PCR data represent the mean of three independent experiments±s.e.m., *P<0.05, **P<0.01, ***P<0.001, ****P<0.0001 (unpaired Student's *t*-test). Daudi and BL41 cells were obtained from the American Type Culture Collection (Teddington, UK) and grown in RPMI-1640 medium (Lonza, Basel, Switzerland; supplemented with 10% (v/v) fetal bovine serum (Invitrogen, Paisley, UK) and 2 mM l-glutamine (Lonza)). Cell lines were sent to LGC Standards for authentication by short tandem repeat profiling.

Although these data indicated that Bach2 expression is regulated by c-Rel, Bach2 has not been previously described as a direct NF-κB target gene. To address this, we analysed chromatin immunoprecipitation sequencing (ChIP-Seq) data from the Epstein–Barr-virus-transformed human lymphoblastoid B-cell line GM12878.^41^ This revealed that the Bach2 promoter is bound by c-Rel together with the other NF-κB subunits, RelA, RelB and p52 (Figure 4a). Moreover, further analysis of ChIP-Seq data obtained for the RelA NF-κB subunit by the Encode consortium confirmed that Bach2 is an NF-κB target gene in multiple B-cell lines (not shown). Consistent with these data, analysis of the human Burkitt lymphoma cell line Daudi, where NF-κB subunits had been depleted by CRISPR/Cas9 mutagenesis, revealed that loss of either c-Rel or RelA reduced Bach2 mRNA levels (Figures 4b and c). However, no effect on Bach2 protein level was seen (not shown) suggesting functional compensation between c-Rel and RelA in these cells, as has been reported previously for these subunits.^42^ Treatment of Daudi cells with the IκB kinase-β inhibitors BMS 345541 or TPCA-1, which inhibit the classical NF-κB pathway and so target both RelA and c-Rel, did result in loss of both Bach2 mRNA and protein (Figures 4d and g), and similar results were seen in the Burkitt cell line BL41 treated with TPCA-1 (Figures 4h and i).

### The role of c-Rel in B-cell lymphoma

Given the large number of studies indicating tumour-promoting roles for c-Rel in lymphoma,^2, 3, 4, 5, 6, 21, 22, 23, 24, 25, 26, 43^ our results showing earlier onset of disease in c-Rel mutant mice were surprising. However, a number of *in vitro* studies have, in addition to their known tumour-promoting activities, revealed tumour suppressor functions for NF-κB subunits.^1^ Moreover, previous reports using mouse models of c-Myc-driven lymphoma have demonstrated that through induction of therapy-induced senescence, NF-κB can function as a tumour suppressor in this context.^27, 28^ Importantly, previous studies of the role of c-Rel in lymphoma have used either patient cells or established laboratory cell lines. In both cases, by analysing 'end-stage' cancer cells, these investigations will have focused on the antiapoptotic effects of NF-κB, which we also see, but will have missed any more complex roles that might occur during the process of lymphomagenesis itself. Our study has therefore allowed the description of a previously unknown role for c-Rel in the prevention of B-cell lymphoma development by regulating the expression of Bach2. However, NF-κB regulation of Bach2 is not restricted to c-Rel and our data also support a role for RelA. Interestingly, in E*μ-Myc* mice RelA regulation of Bach2 was only seen in the 'end-stage' lymphomas (Figures 3h and i), suggesting that c-Rel is the primary driver of Bach2 expression. Nonetheless, this demonstrates the complex interplay between NF-κB subunits, as well as the potential for stage-specific regulation of gene expression during lymphomagenesis. It will be of interest to see if c-Rel can also contribute to the regulation of NF-κB-dependent senescence reported in E*μ-Myc* lymphoma cells.^27, 28^

Bach2 is a transcription factor and known as B-cell tumour suppressor. Interestingly, a recent report illustrated that Bach2 is required for c-Myc-dependent induction of p53 in pre-B cells.^39^ Moreover, loss of Bach2 is associated with the development of pre-B acute lymphoblastic leukaemia.^39^ Bach2 promoter activity is also reduced upon BCR-ABL expression in chronic myeloid leukaemia, through regulation by the transcription factor, Pax5, suggesting that suppression of Bach2 may contribute to lymphoid blast crisis in chronic myeloid leukaemia.^44^ Although we cannot rule out contributions from the other c-Rel-regulated genes we identified, we propose that induction of Bach2 expression by c-Rel/NF-κB provides one mechanism that allows these factors to function as tumour suppressors in the early stages of B-cell lymphoma development. However, some reports have suggested that Bach2 may also contribute towards malignancy in some contexts.^45^ Since the tumour suppressor functions of Bach2 are associated with p53, it is possible that p53 loss or mutation is also the trigger for a change in Bach2 function. Therefore, the consequences of NF-κB regulation of Bach2 expression may vary depending on the stage of lymphoma development.

### Accession numbers

NF-κB ChIP-Seq data sets have been published^41^ (gene expression omnibus, accession code GSE55105).

Microarray data have been submitted to ArrayExpress, accession code E-MTAB-2774.
